# A fast recoiling silk-like elastomer facilitates nanosecond nematocyst discharge

**DOI:** 10.1186/s12915-014-0113-1

**Published:** 2015-01-16

**Authors:** Anna Beckmann, Senbo Xiao, Jochen P Müller, Davide Mercadante, Timm Nüchter, Niels Kröger, Florian Langhojer, Wolfgang Petrich, Thomas W Holstein, Martin Benoit, Frauke Gräter, Suat Özbek

**Affiliations:** Department of Molecular Evolution and Genomics, University of Heidelberg, Centre for Organismal Studies, Im Neuenheimer Feld 329, 69120 Heidelberg, Germany; Heidelberg Institute for Theoretical Studies, Schloss-Wolfsbrunnenweg 35, 69118 Heidelberg, Germany; Kirchhoff Institute for Physics, Heidelberg University, Im Neuenheimer Feld 227, 69210 Heidelberg, Germany; Dioptic GmbH, Bergstraße 92A, D-69469 Weinheim, Germany; Applied Physics and Center for NanoScience, Ludwig Maximilian University, Amalienstr. 54, 80799 München, Germany

**Keywords:** Hydra, Nematocyst, Elastomer, Molecular dynamics, Single molecule force spectroscopy

## Abstract

**Background:**

The discharge of the Cnidarian stinging organelle, the nematocyst, is one of the fastest processes in biology and involves volume changes of the highly pressurised (150 bar) capsule of up to 50%. Hitherto, the molecular basis for the unusual biomechanical properties of nematocysts has been elusive, as their structure was mainly defined as a stress-resistant collagenous matrix.

**Results:**

Here, we characterise Cnidoin, a novel elastic protein identified as a structural component of *Hydra* nematocysts. Cnidoin is expressed in nematocytes of all types and immunostainings revealed incorporation into capsule walls and tubules concomitant with minicollagens. Similar to spider silk proteins, to which it is related at sequence level, Cnidoin possesses high elasticity and fast coiling propensity as predicted by molecular dynamics simulations and quantified by force spectroscopy. Recombinant Cnidoin showed a high tendency for spontaneous aggregation to bundles of fibrillar structures.

**Conclusions:**

Cnidoin represents the molecular factor involved in kinetic energy storage and release during the ultra-fast nematocyst discharge. Furthermore, it implies an early evolutionary origin of protein elastomers in basal metazoans.

**Electronic supplementary material:**

The online version of this article (doi:10.1186/s12915-014-0113-1) contains supplementary material, which is available to authorized users.

## Background

Elastic mechanisms in animals are highly diverse and involve either single rapid movements as in jumping froghoppers and many vertebrates [1] or rhythmic movements as in flying insects [2]. The molecular springs involved in elastic movements are as diverse but have in common unstructured domains that lose conformational entropy upon stretching, generating the restoring force, which finally drives the elastic movement. Although elastic mechanisms are well studied in arthropods, there are few data for lower metazoans and the evolutionary origin of elastic proteins.

**Figure 1:**
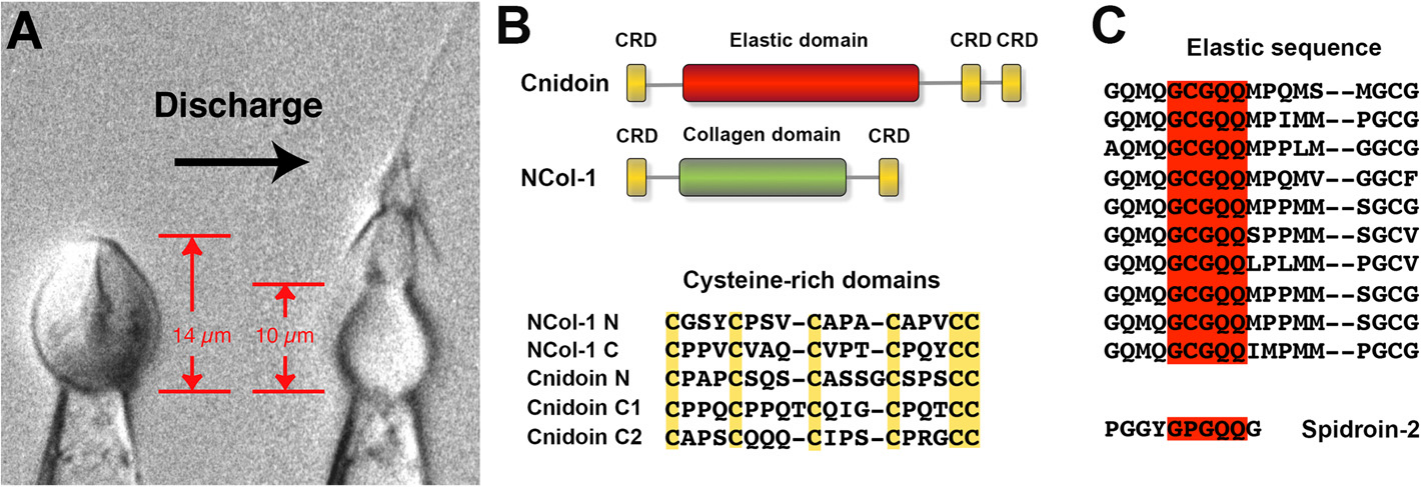
**Capsule recoil after nematocyst discharge and sequence elements in Cnidoin primary structure. (A)** capsule size change during discharge of a stenotele. **(B)** domain organisation of Cnidoin and Minicollagen-1 (NCol-1) and alignment of respective CRD domains. Conserved cysteines are highlighted. **(C)** alignment of the elastic domain of Cnidoin with a homologous sequence in Spidroin-2. Highlighted is the GxGQQ motif present in both sequences.

Nematocytes or stinging cells of jellyfish and other cnidarians produce a unique toxic organelle consisting of a spherical capsule to which a long tubule is attached [3,4]. The tubule is tightly coiled inside the capsule matrix and expelled in a harpoon-like fashion during a nanosecond discharge process [5]. By the synthesis of poly-γ-glutamate at final maturation, nematocysts in hydrozoans are charged with an osmotic pressure of about 150 bars [6,7]. Prior to discharge, the capsule volume is increased by 30% due to osmotic swelling [5,8] and the explosive exocytosis releases the kinetic energy stored in the elastically stretched capsule wall with an extreme acceleration of 5,410,000 g [5]. After discharge, the size of the capsule is reduced to about 50% (Figure 1A) [8,9].

The nematocyst wall is built of a dense matrix consisting mainly of minicollagens, which represent a unique feature of cnidarians [10-12]. Minicollagens constitute a large protein family in *Hydra* and share a common domain organisation comprising a short central collagen triple helix flanked by variable polyproline stretches and cysteine-rich domains (CRDs) supposed to be involved in network formation [4,13,14]. While minicollagens can account for the high tensile strength required for the capsule wall to withstand a pressure of 150 bars [11], elastomeric proteins, which can store the energy for the extraordinarily fast kinetics of discharge, are unknown.

Recently, we have presented the proteome of *Hydra* nematocysts, which showed an extracellular matrix-like composition of the nematocyst supra-structure [14]. A novel component of the insoluble nematocyst shell was designated Cnidoin, a protein with a central glycine-glutamine-rich sequence flanked by minicollagen CRDs (Figure 1B). The highly repetitive central domain is homologous to the glycine-rich sequence of the spider silk protein Spidroin-2 [15]. A conserved motif in both proteins is GXGQQ, where X is cysteine in Cnidoin and proline in Spidroin-2 (Figure 1C). Cnidoin protein has been shown to be co-localised with minicollagens in the nematocyst wall, suggesting a mechanical function in nematocysts [14].

It is well accepted that disordered peptides such as Spidroins, the PEVK segment in titin, or resilin in the muscle of fly wings can serve as elastic elements in protein structures and materials [16-18]. Elongation of such an unstructured peptide is entropically unfavoured and involves rupturing of non-specific interactions of hydrophobic or electrostatic nature along the chain. Consequently, this process requires significant mechanical force, resulting in high elasticity. The elastic features of disordered peptides as defined by their amino acid sequence have been documented by both experimental atomic force microscopy (AFM) studies and molecular dynamics (MD) simulations, with remarkable agreement [16,19-21].

Here, we provide evidence for an elastomeric function of Cnidoin in nematocyst morphogenesis and discharge. As with minicollagens, Cnidoin is expressed exclusively in developing nematocytes in the body column of *Hydra*. We demonstrate a concomitant incorporation of Cnidoin and minicollagens into both the wall and tubule structures of all nematocyst types. Recombinantly expressed and purified Cnidoin protein has a high tendency for aggregation and forms amorphous sheets and fibres as described for spider silk proteins. MD simulations of the repetitive elastic sequence of Cnidoin suggest an elastic behaviour comparable to other elastomeric proteins, such as the silk disordered domain or resilin. The predicted molecular elasticity is in line with force spectroscopy measurements on single Cnidoin molecules. Our data provide an explanation for the unusual biomechanical properties of the Cnidarian nematocyst at the molecular level and give insights into the evolution of elastomeric proteins.

## Results

### Primary structure and domain organisation of Cnidoin

A full-length clone for Cnidoin was isolated from *Hydra magnipapillata* cDNA based on the predicted gene sequence annotated in our recent proteome analysis [14]. The primary structure comprises a signal peptide (1–21) and an unusually large putative propeptide (22–111) terminating at a basic Lys-Arg dipeptide sequence, which is conserved in diverse nematocyst proteins (see Additional file 1: Figure S1A). The predicted mature protein contains 403 amino acids and has a calculated molecular weight of 41.6 kDa. The central part of the protein comprises an extended sequence rich in glycine and glutamine residues whose repetitive motif QMQGCGQQXP (X is mostly methionine) shows high similarity to the elastic glycine-rich sequence of the major spider silk component from *Nephila clavipes*, Spidroin-2 [22] (Figure 1C). The amino acid composition of the elastic motif in Cnidoin is unusual in containing a high percentage of methionine and cysteine (see Additional file 1: Figure S1B). At both termini Cnidoin contains short CRDs homologous to those in canonical minicollagens as minicollagen-1 (NCol-1) [23], suggesting a possible linkage to the minicollagen network via intermolecular cysteine links (Figure 1B). The Cnidoin primary sequence does not contain N-glycosylation sites, but several potential sites for O-glycosylation. Treatment of nematocyst preparations with N- and O-glycosidases, however, did not result in a molecular weight shift ruling out posttranslational modifications by sugars (data not shown).

### Cnidoin is expressed in developing nematocyst nests and co-localises with minicollagens

**Figure 2:**
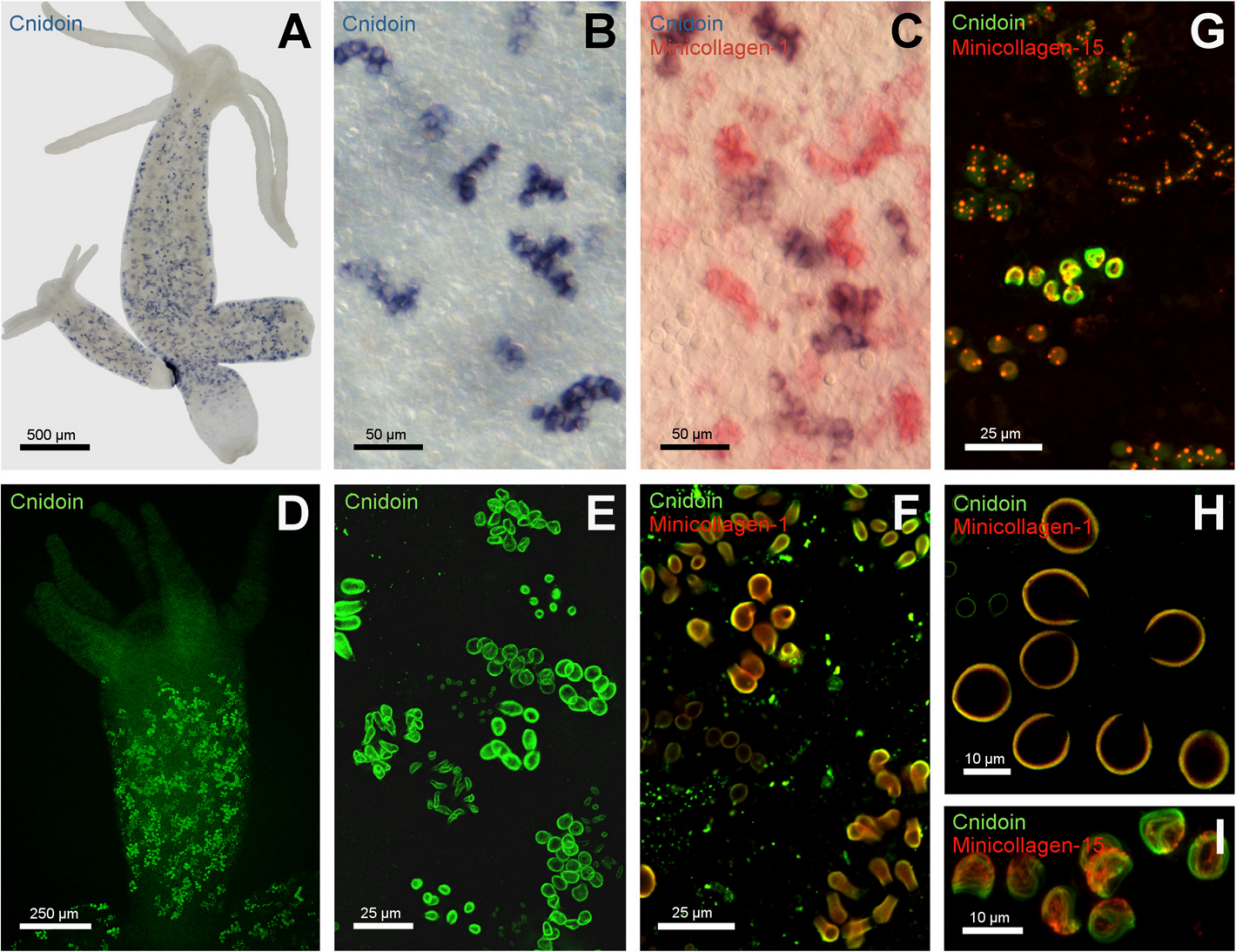
**Gene expression pattern and immunohistochemical localisation of Cnidoin. (A)** gene expression pattern as shown by *in situ* hybridisation. The signal is detected in nests of developing nematocytes in the body column. **(B)** close-up of nematocyte nests shown in A. **(C)** double *in situ* hybridisation of Minicollagen-1 (red) and Cnidoin (blue). **(D)** Antibody staining reveals nests of developing nematocytes in the body column of *Hydra*. **(E)** enlarged view of the immunostaining shown in D. **(F)** costaining of Minicollagen-1 and Cnidoin in PFA-fixed animals. Both signals localise to the capsule wall. **(G)** co-staining of Minicollagen-15 and Cnidoin in Lavdovsky-fixated animals. This fixation reveals the presence of both proteins in the tubule of developing nematocysts. **(H)** enlarged view of a nest with Cnidoin and Minicollagen-1 immunostaining. **(I)** magnification of a nest with a tubule-specific signal for Cnidoin and Minicollagen-15. PFA, paraformaldehyde.

Whole mount *in situ* hybridisation (ISH) experiments were carried out to determine the expression pattern of the *Cnidoin* gene. As shown in Figure 2A the expression of *Cnidoin* is restricted to developing nests of nematocytes in the body column of *Hydra*. Tentacles, head and peduncle regions were free of signals. This pattern is highly reminiscent of other nematocyst-specific structural genes, such as minicollagens or NOWA [24,25]. As shown in Figure 2B, mostly late stages of developing capsules with a pronounced nematocyst vesicle showed a Cnidoin signal. In nests of early developmental stages the signal was not detectable probably due to lower expression rates (data not shown). When double ISH experiments using Cnidoin and NCol-1 probes were performed, the signals were mostly co-localised in developing nematocyte nests (Figure 2C).

To localise Cnidoin protein during capsule development, whole mount immunostainings were performed using a polyclonal Cnidoin antibody raised against the C-terminal CRD. Nematocytes in *Hydra* develop in nests of 8 to 32 cells that originate from interstitial stem cells (i-cells). Their morphogenesis involves the continuous secretion of proteins into a growing post-Golgi vesicle. Capsule maturation is marked by nematocyte separation into single cells that migrate to the tentacles to be incorporated into battery cells. Immunostainings using paraformaldehyde (PFA) fixation showed Cnidoin in developing nematocyst nests of all stages and capsule types in the body column of *Hydra* (Figure 2D-E). The staining pattern is again highly reminiscent of minicollagens that lose antigenicity during capsule maturation [24,25]. Co-staining using minicollagen-1 antibody revealed a co-localisation of the two proteins to a large extent in the developing capsule walls (Figure 2F, H). In contrast to PFA fixation, Lavdovsky fixated animals showed in addition tubule staining patterns in developing nests, which co-localised with the tubule-specific minicollagen-15 (Figure 2G, I). This observation might be due to an altered molecular arrangement of Cnidoin in the different capsule parts. The loss of Cnidoin staining in the tentacles indicates a tight incorporation of the protein into the collagenous wall and tubule structures as already suggested by the possession of CRDs.

### Recombinant Cnidoin is highly hydrophobic and spontaneously forms fibrous aggregates

**Figure 3:**
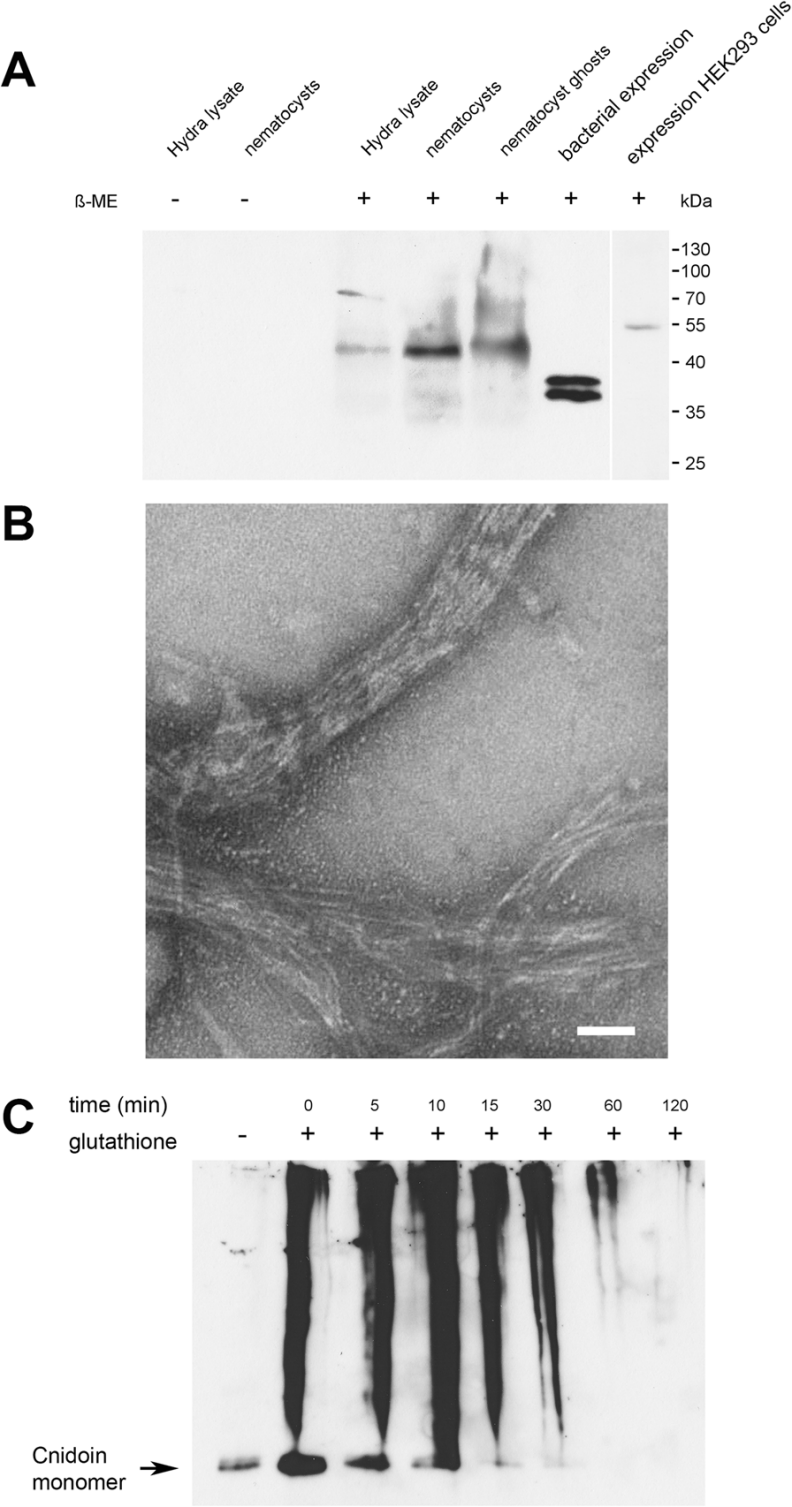
**Western blot detection and self-aggregation of Cnidoin. (A)** western blot analysis of Cnidoin in isolated nematocysts, the insoluble fraction of nematocysts (ghosts), whole hydra lysate, and after recombinant expression in bacteria and HEK293 cells. **(B)** transmission electron micrograph of recombinant Cnidoin forming bundles of fibres. Scale bar is 100 nm. **(C)** western blot analysis of recombinant Cnidoin induced to form disulphide-linked polymers by glutathione treatment.

In western blot analysis Cnidoin was detected as a single band of about 42 kDa in hydra lysates, isolated nematocyst capsules as well as in nematocyst ghosts (Figure 3A). This is slightly lower than the calculated molecular mass of 51.6 kDa including the putative propeptide, indicating that propeptide cleavage does occur during secretion into the nematocyst vesicle. When expressed recombinantly in HEK293 cells, full-length Cnidoin exhibited a molecular mass of about 53 kDa, which matches the calculated mass of Cnidoin including a C-terminal his-tag (53.1 kDa) (Figure 3A). We conclude that in HEK293 cells propeptide cleavage does not occur resulting in the observed molecular mass difference to the native protein, which accounts for the propeptide fragment. The western blot signal was exclusively detectable under reducing conditions suggesting an incorporation of Cnidoin into the disulphide-linked capsule wall polymer similar to NCol-1 and NOWA [13,26].

When full-length Cnidoin was expressed in bacteria the protein formed highly insoluble aggregates that were already detectable as large pellets after cell lysis. Purification of Cnidoin via histidine tag was, therefore, performed under denaturing conditions using 8 M urea, which led to transient solubilisation of the protein aggregates. Cnidoin was essentially insoluble in physiological aqueous buffers. The recombinantly expressed protein exhibited a lower apparent molecular mass of about 38 kDa and a prominent double band, probably indicative of altered migration behaviour by intramolecular disulphide bonding. Purified Cnidoin spontaneously formed macroscopic fibrillar structures in 8 M urea when cooled samples were gradually brought to room temperature [14]. This coacervation process is reminiscent of elastin-derived peptides where spontaneous self-assembly of monomers is induced by temperature increase depending on protein and salt concentration [27]. The driving force for coacervation, which precedes microfibrillar deposition and cross-linking in elastin, are hydrophobic domain interactions in an aqueous environment. Analysis of Cnidoin fibres by transmission electron microscopy revealed bundles of linear fibres in the nanometre range reflecting the common filamentous nature of elastomeric proteins (Figure 3B). Due to the presence of terminal minicollagen CRD domains we hypothesised that Cnidoin polymerisation was dependent on disulphide coupling. This was demonstrated by glutathione treatment of recombinant Cnidoin samples, which showed gradual formation of high molecular weight oligomers in non-reducing SDS-PAGE (Figure 3C). A similar behaviour is observed for recombinant NCol-1 samples [13], suggesting a possible copolymer formation of both proteins. Our hypothesis was further strengthened by incubating recombinant NCol-1 with increasing amounts of Cnidoin in the presence of glutathione, which resulted in a dose-dependent increase of disulphide-linked polymer formation (see Additional file 2: Figure S2).

### Cnidoin shares elastomeric functions with other disordered proteins

The sequence composition of Cnidoin (see Additional file 1: Figure S1B) with a high glycine and proline content of 24% and 10%, respectively, shows typical features of disordered proteins. Intrinsically unstructured proteins have been classified previously as sequences with low overall hydrophobicity and high net charge as compared to structured proteins [28,29]. These two properties are believed to prevent the formation of a solvent-inaccessible protein hydrophobic core typically formed by proteins with a well-defined structure. However, with a high mean hydrophobicity of 0.48 and a low mean net charge of 0.007, Cnidoin falls into the region known to be covered by natively folded proteins. Nevertheless, the high glycine and proline content impedes the formation of a native structure, as predicted by DisEMBL (see Additional file 3: Figure S3A) [30]. While the high hydrophobicity in this case does not result in protein core formation, it gives rise to the observed high propensity for self-aggregation (compare Figure 3B). Again, Spidroin-2 follows similar tendencies with a (slightly lower) mean hydrophobicity of 0.39, a mean net charge of 0.0002 and a proline content of 8% to 15%.

We investigated the degree of ordering additionally by using mid-infrared spectroscopy following the considerations of Byler and Susi [31], who showed that disordered proteins such as casein exhibit a low number of spectral components in the so-called Amide I band of the mid-infrared spectrum. Furthermore, both the existence and the above-average width of a peak around 1,645 cm^−1^ (that is, around a wavelength of 6.08 μm) were considered as an indication of a low degree of ordering. The number of spectral components of casein together with the corresponding numbers for other standard proteins investigated is shown in Additional file 3: Figure S3B [31]. The optimum number of components in Cnidoin tended to be low in relation to standard proteins known to be highly ordered [31] and comparable to that of casein. Moreover, Cnidoin does exhibit a broad peak around 1,645 cm^−1^ (see Additional file 3: Figure S3C). In this sense, our spectroscopic data and its analysis support the hypothesis of the disordered nature of Cnidoin.

**Figure 4:**
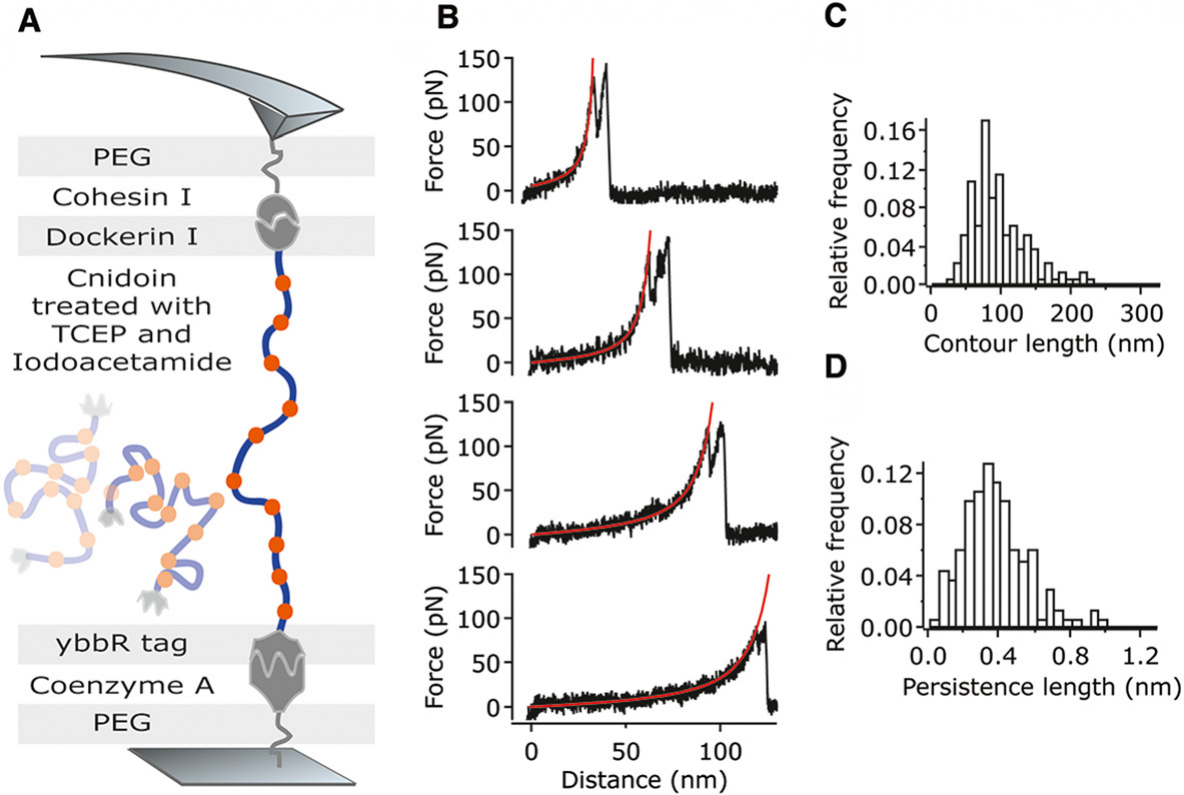
**AFM force spectroscopy on recombinant Cnidoin monomers. (A)** Schematic diagram of the AFM force spectroscopy experiment on single Cnidoin molecules, containing several cysteins with thiol groups (orange dots). For breaking disulphide bonds and preventing their reformation, Cnidoin was treated with TCEP and iodoacetamide. **(B)** Typical force-distance traces of Cnidoin displaying a final double peak, which is characteristic of the cohesin-dockerin rupture [1]. Force-distance traces show characteristic worm-like chain (WLC) behaviour and lack further pronounced features, corroborating the disordered nature of Cnidoin molecules. For quantification of contour and persistence length, a WLC model was fitted to the data (red line). **(C)** Histogram of the contour length distribution of stretched Cnidoin, yielding an average contour length of 94 nm and a standard deviation of 43 nm. **(D)** Histogram of the persistence length distribution of Cnidoin, yielding an average persistence length of 0.37 nm and a standard deviation of 0.23 nm. AFM, atomic force microscopy; TCEP, (tris(2-carboxyethyl)phosphine).

To further experimentally confirm the sequence-based prediction of high disorder in Cnidoin, we performed AFM single molecule force spectroscopy using a Cnidoin construct with a C-terminal type I dockerin tag and an N-terminal ybbR tag (Figure 4A). While the ybbR tag allowed for attaching the construct covalently to a coenzyme A surface, the dockerin tag enabled specific non-covalent binding of the construct to a type I cohesin functionalised cantilever tip (see Additional file 4: Figure S4). By retracting the tip from the surface, Cnidoin molecules were stretched via the C-terminal dockerin tag while force-distance traces were recorded. As previously shown in AFM experiments, forced dissociation of the type I cohesin-dockerin interaction preferentially goes along with a characteristic double peak of 8 nm separation in contour length space [32]. This characteristic feature was used as a positive indicator for a specific stretching event (For evaluation of discarded curves see Additional file 5: Figure S5). Force-distance traces of Cnidoin molecules showed characteristic worm-like chain (WLC) behaviour without any further pronounced features (Figure 4B). The latter would indicate conformational rearrangements, as seen in stretching of structured proteins [33]. The lack of such features in force-distance traces of Cnidoin corroborates the disordered nature of the protein. Fitting a WLC model yields a distribution of both contour (Figure 4C) and persistence length (Figure 4D). Within a standard deviation of 43 nm, the average contour length of 94 nm is in good agreement with the estimated contour length of 110 nm (283 amino acids at an extension of 0.32 nm per amino acid plus an overall linker length of 20 nm including the polyethylene glycol (PEG) linker and the cellulose-binding module (CBM)-cohesin-dockerin anchor). The slight shift to smaller contour lengths may result from non-specific adsorption of Cnidoin molecules to the surface. Because of the harsh disulphide treatment, oppressing intermolecular bonds, only a minor fraction of curves yield contour lengths significantly larger than expected. Importantly, the featureless WLC characteristic of force-distance traces does not vary with the contour length, nor does the persistence length. The persistence length, which measures the apparent flexibility of a polymer against entropic force, yields an average of 0.37 ± 0.23 nm. This value matches the persistence length of 0.4 nm, which is frequently assumed for stretching unfolded proteins at similar force ranges in AFM experiments [33-35]. The standard deviation of the persistence length results predominantly from intrinsic uncertainty of the zero force in AFM experiments. In conclusion, the featureless and WLC like force-distance traces of Cnidoin measured by AFM confirm the high disorder of the protein’s structure.

**Figure 5:**
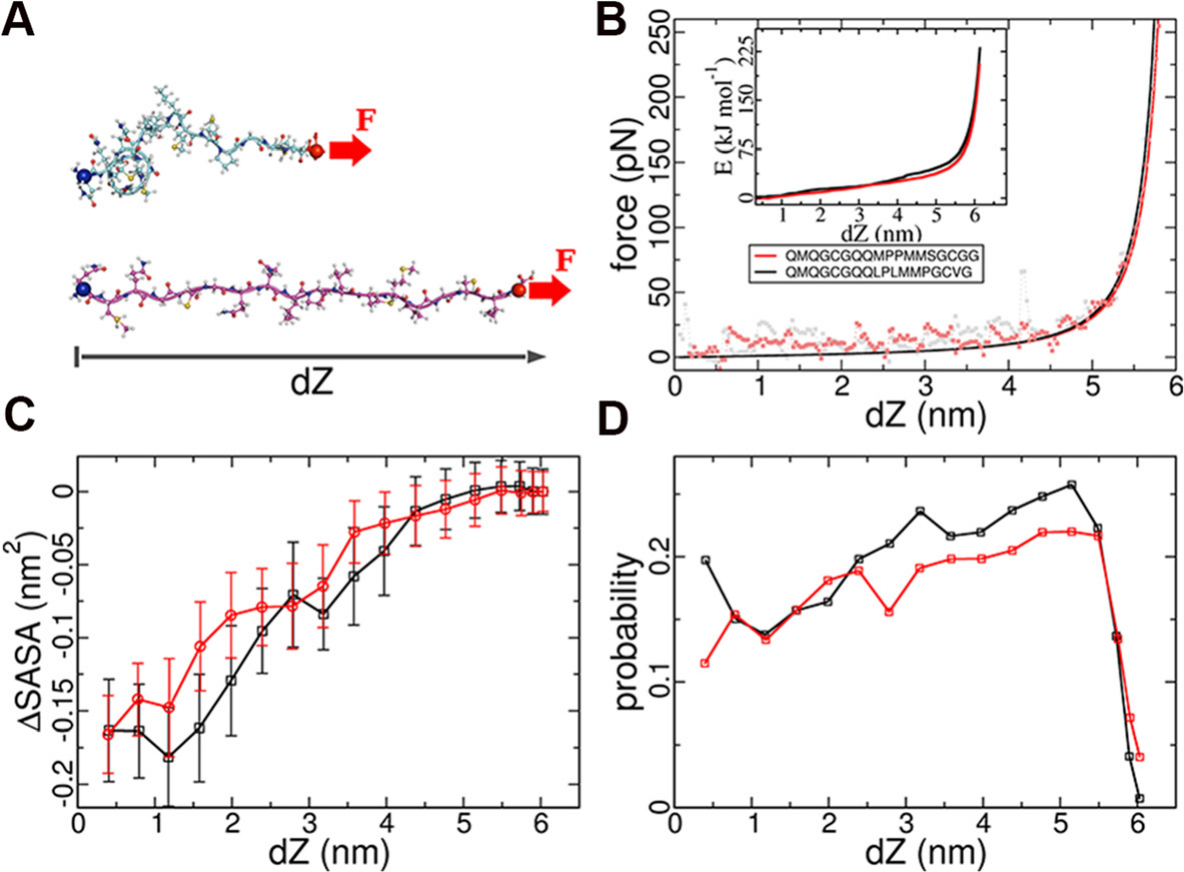
**Molecular elasticity of Cnidoin peptides from MD simulations. (A)** two representative (collapsed and extended) conformations of a Cnidoin peptide unit. To obtain force-extension curves, N-terminal C-alpha atoms (blue spheres) were fixed, while the C-termini (red spheres) were subjected to a force acted along the extension (red arrows). **(B)** mean forces calculated from umbrella sampling. The force profiles of two different Cnidoin repeat units (squares) were fitted with the worm-like chain model (solid lines). Resulting free energy profiles along the end-to-end distance are shown in the inset. **(C)** residue-averaged hydrophobic surface burial of two Cnidoin peptides measured by disappearance of solvent accessible surface area (ΔSASA). (**D**) PPII conformation content in the Cnidoin peptides along peptide extension. **(C)** and **(D)** use the same colour code as **(B)**. MD, molecular dynamics; PPII, polyproline II.

To complementary assess the elasticity of Cnidoin, we calculated the force-extension profile of two representative repeat units of Cnidoin using umbrella sampling [20]. To this end, Cnidoin peptide conformations were extensively sampled at varying end-to-end distances dZ (Figure 5A). Resulting average resisting forces against Cnidoin extension are shown in Figure 5B, with the resulting free energy shown in the inset. We observed a force plateau followed by a steep increase in force at larger extensions. The mean forces were fitted by the WLC model (solid line in Figure 5B), [36], which predicted persistence lengths of 0.89 ± 0.1 and 0.66 ± 0.06 nm for the Cnidoin repeat units shown in Figure 5B in red and black, respectively. Therefore, Cnidoin is found to be as elastic as silk disordered peptides, which have formerly been reported to have a persistence length of 0.74 nm in MD simulations in the force range probed here [20]. Hence, the MD simulations confirm the finding from AFM experiments of a featureless force-extension curve resembling a disordered protein such as silk. We note that for both silk [20,37] and Cnidoin investigated here, the persistence length from simulations is higher than in experiments, although the second simulated Cnidoin repeat unit lies within the experimental range (Figure 5B, red line).

As demonstrated in Figure 3, Cnidoin has a strong tendency for aggregation, which might be additionally enhanced by intermolecular disulphide links in the elastic domain. In order to assess Cnidoin in the biological environment of mature nematocysts, we resorted to MD simulations of single Cnidoin fragments and of a Cnidoin fragment embedded into a bundle of fragments, respectively. In agreement with the prediction of high disorder from sequence and the absence of forced unfolding events in AFM experiments (Figure 4B-D), Cnidoin peptide units did not form stable secondary structures in our simulations. The secondary structure of two Cnidoin peptides was monitored during 500-ns equilibrium MD simulations by DSSP: Dictionary of Secondary Structure for Proteins [38]. The peptides were found to form coils, bends and turns as well as a polyproline-II type structure (see below), all of which are typical backbone propensities of disordered peptides. The sequence motif *GCGQQ* was found to exist as a turn, and thus structurally related, but, due to the missing proline, not identical to the π-helix formed by the silk protein motif *GPGQQ* [39].

A mean force of 12.7 ± 0.7 pN was found to dominate at low extensions between 1.0 to 3.5 nm of single Cnidoin peptides. This mean resisting force results from both rupturing non-specific interactions along the chain and also entropic effects. We find the burial of hydrophobic side-chains from water to be one of the key driving factors that generate resisting forces against extension of Cnidoin. As shown in Figure 5C, the hydrophobic surface of Cnidoin buried during collapse is 0.17 ± 0.03 nm^2^ per amino acid, as measured by the decrease in hydrophobic solvent accessible surface area (ΔSASA). This hydrophobic surface burial is similar in magnitude to the related silk peptides, and one of the highest among reported disordered proteins [20]. Together with the extraordinarily high mean hydrophobicity of these two types of disordered proteins, Cnidoin and silk have been apparently designed to self-assemble into larger aggregates driven by the hydrophobic effect. Polyproline II (PPII) conformation, an extended peptide conformation frequently observed in high proline-content peptides, was also monitored as a function of peptide extension [40]. As depicted in Figure 5D, the PPII content in the Cnidoin peptide increased with stretching as observed for other disordered proteins previously [20] and is another factor contributing to the resistance against extension and, thus, to the elasticity. We find the stretching of Cnidoin molecules within a bundle of Cnidoin proteins to give rise to higher force-extension curves (plateau at approximately 400 pN). Fitting yields an apparent persistence length of approximately 0.1 *±* 0.003 nm, (see Additional file 6: Figure S6) which is lower than the one obtained for single Cnidoins (Figure 5B), reflecting a stronger coiling propensity. Thus, in the context of mature nematocysts, intermolecular interactions of similar nature as those discussed here for a single peptide can give rise to a further increase of the apparent elasticity of Cnidoin in bundles or networks.

**Figure 6:**
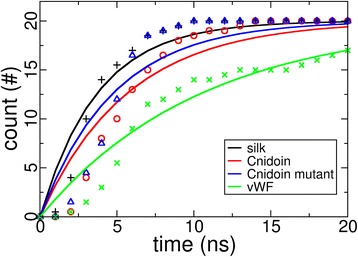
**Cumulative successful collapsing events of four disordered proteins.** Each data set contains 20 independent collapse simulations along with an exponential fit (solid line) to determine the lifetime of the extended state.

The hypothesis that the shortening of Cnidoin peptides drives the extremely fast discharge of nematocysts has been confirmed by the observation that Cnidoin is equipped with the elasticity required to drive such a discharge. Fast collapse dynamics of Cnidoin would be required given the short sub-microsecond time scale of discharge [5]. To test the collapse dynamics, highly stretched conformations of the two repetitive Cnidoin peptide units were subjected to force-quench simulations to monitor collapse dynamics in comparison to other disordered peptides, namely two silk amorphous peptides and two domain linker peptides in the von Willebrand Factor (vWF). Both are also disordered peptides but with sequence compositions similar to folded proteins (see Additional file 7: Figure S7) [17,41]. The number of trajectories in which a fully stretched peptide has reached the collapsed state is shown in Figure 6, from which the lifetime of the extended state was calculated. The Cnidoin peptide was found to have a lifetime of 5.7 ns, which is longer than that of silk (3.7 ns) and shorter than that of the vWF linker (10.5 ns). Thus, Cnidoin has a moderate collapse dynamics between the highly elastic silk protein and the vWF linker, for which an elastomeric function is unknown. The lifetime of extended mutated Cnidoin peptides lacking methionines was found to be 4.7 ns, suggesting that the large methionine side chains do not favour fast collapse. The high content of methionine residues in Cnidoin thus is likely to have a function different from enhancing collapse.

## Discussion

Elastomeric proteins confer the biomechanical properties for fibres and matrices that undergo reversible and repetitive deformation [42]. They have evolved in a diverse range of animals and often fulfil highly specialised biological functions as in lung alveoli of higher vertebrates, wing joints of flying insects or in spider silk [43]. Although exhibiting a broad range of sequence variations, rubber-like elastomeric proteins share common properties in combining repetitive, highly disordered sequence elements with cross-linking motifs. In addition, most elastomeric proteins undergo spontaneous self-assembly to polymers by their hydrophobic nature [43].

**Figure 7:**
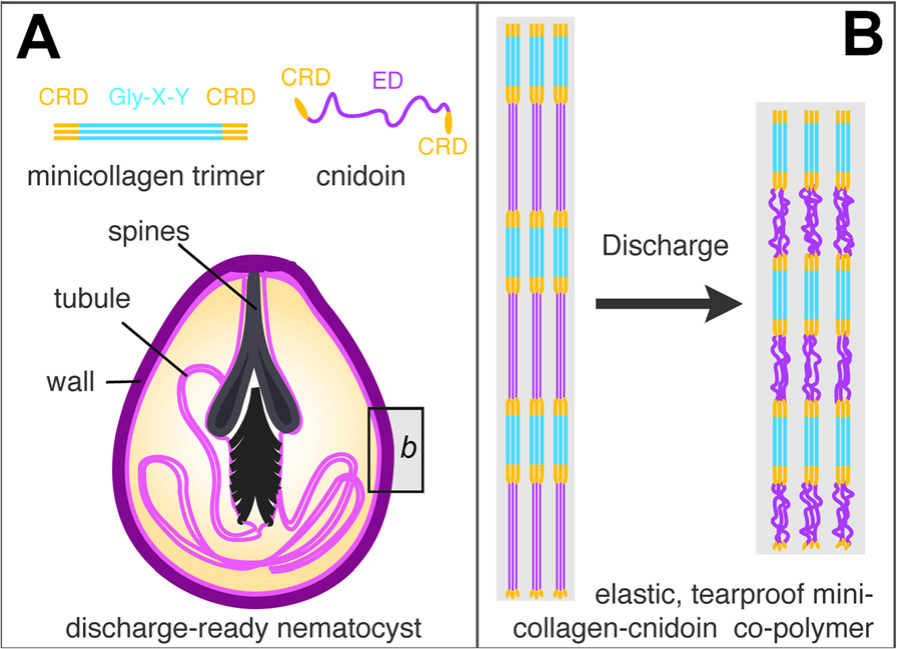
**Model of elastic nematocyst discharge. (A)** schematic representation of nematocyst architecture and minicollagen and Cnidoin components (capsule redrawn from [4]). **(B)** model of the elastic, tear-proof minicollagen-Cnidoin copolymer facilitating nematocyst discharge.

Our combined experimental and computational study suggests that Cnidoin shares elastomeric functions with other disordered proteins. Having a similar persistence length with silk protein, the Cnidoin repetitive sequence features high flexibility among unstructured peptides. The extension of Cnidoin requires a noticeable mean force of around 12 pN for extensions between 10% and 70% of the contour length. We believe this high elasticity confers the high internal pressure of the nematocyst and agrees with the mechanical work upon collapsing Cnidoin with an apparent persistence length of 0.4 nm from 50% of its contour length to a fully collapsed state. This scenario brings the molecular measurements in line with the macroscopic mechanical observations, supporting the notion of a pressurised nematocyst that comprises a wall of assembled and highly extended elastomeric Cnidoin cross-linked by largely inextensible minicollagen and possibly disulphide bonds (Figure 7).

The biological function of the remarkably high methionine content of 17% in Cnidoin is intriguing. As the metabolic costs for methionine synthesis are the highest among all amino acids [44], rendering it a rare residue in proteins on average, and yet methionines do not favour fast peptide collapse nor feature outstanding hydrophobicity, they should contribute to the mechanics of nematocysts in a distinct way. The sulphur atom in methionine side-chains might be used as an additional cross-linking point, for example, via thioether bonds, in the peptide matrix of the nematocyst wall, which is subject to further investigations.

Apart from the extraordinary methionine and cysteine content, Cnidoin exhibits an overall amino acid composition, which on first sight is contradictory to its mechanical function. Most disordered proteins feature a lower hydrophobicity and higher net charge than natively folded proteins, which is key to abstaining from forming a well-structured protein hydrophobic core. However, here and analogously for silk, the remarkably hydrophobic Cnidoin impedes core formation by a high glycine and proline content and yet drives self-assembly into a robust protein wall withstanding high pressure without leaking.

Interestingly, the high proline content of Cnidoin falls into the region covered by silk spidroin type-2 proteins and is known to be the primary reason for the high extensibility and elasticity of silk fibers [45,46], suggesting that prolines take over the same role in the nematocyst wall.

Cnidoin as a highly extensible and yet robust wall material provides the elasticity for a fast discharging nematocyst. Our computational analysis on single Cnidoins and Cnidoin bundles suggests that the disordered region can gain additional rubber-like elasticity through intermolecular interactions, which may additionally include covalent cross-links between cysteines and possibly methionines. Pressing questions are how numerous these cross-links are in the nematocyst wall, how they can cause a limited order in the network, how they possibly provide additional elasticity, similar to the tyrosine- or lysine-mediated cross-links in the elastomers resilin and elastin, and how they prevent leaking even under extreme pressures.

## Conclusions

Our data show for the first time how the extremely fast and powerful dynamics of nematocyst discharge in *Hydra* can be explained on the molecular level (Figure 7). The extraordinarily high speed of discharge is due to the release of energy stored in the stretched configuration of the minicollagen-Cnidoin copolymer of the capsule wall that is under a very high initial osmotic pressure (Figure 7B). During discharge the capsule collapses and releases the energy (Figure 1A). The mode of copolymer formation as suggested by the possession of homologous CRDs in Cnidoin and minicollagens represents an attractive mechanism for biotechnological approaches as it offers unlimited fine-tuning of the ratio between elastic and stress-resistant components in forming polymers with different mechanical properties. Future research will show how the formation of such polymers might be feasible under controlled conditions.

## Methods

### Animals

*Hydra magnipapillata* was used for all experiments. Animals were cultured in Hydra medium at 18°C and fed two to three times a week with freshly hatched *Artemia salina* nauplii. Animals used for the experiments were starved for 24 hours. Intact nematocysts were isolated from whole Hydra tissue as described by Weber *et al*. [47]. Sucrose (10%) was added to the solutions in order to prevent an osmotically triggered discharge of the nematocysts.

### Isolation of Cnidoin cDNA and construction of expression vector

Preparation of whole RNA was performed using the RNeasy Mini Kit (Qiagen, Venlo, Netherlands) according to the manufacturer’s instructions. The isolated RNA was transcribed by reverse transcriptase into cDNA. Using a pair of primers encompassing the predicted Cnidoin sequence (forward: ATGTCTCGATTACTACTTC, reverse: TTATCTCTTTTTACCAAAAGCTCC), a PCR was performed and the purified PCR product was ligated into the pGEM-T Vector (Promega, Madison, WI, USA) by TA cloning and the sequence was verified by automated sequencing.

Recombinant expression in *Escherchia coli* BL21 (DE3) cells was performed from a pET21b vector, which introduces a C-terminal polyhistidine tag. Cnidoin protein was exclusively found in inclusion bodies and purified under denaturing conditions (8 M urea) using Ni-NTA beads. For force spectroscopy experiments a Cnidoin fragment carrying an N-terminal ybbR-tag and a C-terminal dockerin-histidine-tag was generated by cloning the cDNA fragment coding for amino acid residues 150 to 419 including primer sequences coding for the respective tags into the pet21b vector.

### SDS PAGE and western blot

A polyclonal guinea pig antibody was raised against a peptide encompassing the sequence of the second C-terminal CRD domain (GCAPSCQQQCIPSCPRGCCGA) of Cnidoin (Eurogentec, Seraing, Liège, Belgium). Isolated nematocysts were solubilised by heating (95°C, 10 minutes) in sample buffer with or without 2-mercaptoethanol as indicated in the experiments. Hydra lysate was prepared by dissolving an animal in reducing or non-reducing sample buffer by heating and vortexing. Nematocyst ghosts, the insoluble fraction of nematocysts, were obtained by extended SDS washing of the isolated and discharged capsules. The ghosts were afterwards solubilised in sample buffer. The samples were separated by SDS-PAGE using 12% gels and transferred to nitrocellulose membranes by wet blotting. Blocking was performed for one hour with 5% milk powder in PBS 0.1% Tween20. After three 10-minute washes with 0.5% milk powder in PBS 0.1% Tween20, the membrane was incubated with the Cnidoin antibody (1:1000 in washing solution) for 1.5 hours, followed by three five-minute washing steps. The primary antibody was detected using an anti-guinea pig antibody coupled to horseradish peroxidase (1:1000 in washing solution) for one hour.

For the polymerisation assay, reduced glutathione was added to a final concentration of 1 mM to recombinantly expressed Cnidoin. The protein was incubated at 37°C. Samples were taken at indicated time points, mixed with non-reducing sample buffer and boiled for five minutes at 95°C. Afterwards the samples were kept on ice, until all time points were covered. The samples were separated by a gradient SDS-PAGE (4% to 20%), blotted on nitrocellulose, blocked with 5% BSA and detected by an anti-penta-his antibody.

The copolymerisation of recombinant Minicollagen-1 MBP (maltose binding protein) and Cnidoin was performed in the presence of 10 mM reduced glutathione. Minicollagen-1 MBP was purified by Ni-NTA from HEK293 cell culture supernatant as described [13,26]. Cnidoin was obtained by isolating recombinant protein from *E. coli*. As indicated in the experiment, various amounts of Cnidoin were added to the Minicollagen-1 containing sample in the presence of glutathione. The sample was mixed with non-reducing sample buffer and boiled immediately at 95°C. Separation and detection was performed as described above.

### Immunocytochemistry

*H. magnipapillata* were relaxed in 2% urethane in Hydra medium and then fixed in freshly prepared 4% PFA in hydra medium or Lavdovsky’s fixative (50% ethanol, 10% formaldehyde, 4% acetic acid, 36% water) for thirty minutes or five hours, respectively. The fixative was removed by three 10-minute washing steps with PBS 0.1% Triton X100. The antibody was diluted 1:250 in PBS 1% BSA and incubated overnight at 4°C. For co-stainings with two antibodies, both antibodies (minicollagen-1 1:500, minicollagen-15 1:1000) were incubated simultaneously overnight at 4°C. To remove unbound antibodies three 10-minute wash steps with PBS 0.1% Triton X100 were performed. The incubation with the second antibodies was performed for two hours at room temperature. For detection of Cnidoin, a goat anti-guinea pig Alexa 568 coupled antibody was used, and for Minicollagen-1 and Minicollagen-15 a goat anti-rabbit Alexa 488 coupled antibody. The secondary antibodies were diluted 1:400 in PBS 1% BSA. To remove unbound antibodies, the animals were washed three times with PBS and then mounted on object slides with PBS 90% glycerol.

### *In situ* hybridisation

The *in situ* probes were amplified from the Cnidoin-pGEM-T construct by PCR using the M13 forward and reverse primers. The purified PCR product was transcribed *in vitro* to DIG labelled RNA by Sp6 RNA polymerase (antisense probe), purified by precipitation with ammonium acetate and quality checked on a 1% agarose gel. As negative control, the sense probe was produced in the same way, using the T7 RNA polymerase. The *in situ* probes were diluted to approximately 5 ng/μl and then used in a 1:100 dilution for the experiment. Animals were relaxed with 2% urethane in Hydra medium and fixed overnight with freshly prepared 4% PFA in Hydra medium. The fixed animals were transferred to 100% ethanol and rehydrated in five minute steps using 75%, 50%, 25% ethanol in PBS, 0.1% Tween20 (PBT). After three five-minute washing steps with PBT the animals were incubated with 1× proteinase K in PBT for seven minutes. The reaction was stopped by adding 4 mg/ml glycine in PBT. Then, the animals were equilibrated twice in 0.1 M triethanolamine (TEA) for five minutes and incubated for five minutes each with 0.25% and 0.5% acetanhydride in TEA, followed by two washing steps with PBT. Then, a re-fixation with 4% PFA was performed for 20 minutes at room temperature, followed by five five-minute washing steps with PBT. The animals were incubated with hybridising solution (50% formamide, 5× SSC (0.75 M NaCl, 0.075 M sodium citrate in water, pH 7.0), 1× Denhardt's (0.02% polyvinyl pyrrolidone, 0.02% Ficoll, 0.021% BSA in water), 0.2 mg/ml yeast RNA, 0.1 mg/ml heparin, 0.1% Tween20, 0.1% Chaps, 10% H_2_O) for 10 minutes and then pre-hybridised in hybridising solution for two hours at 55°C. The probes were diluted in hybridising solution and denatured by heating (75°C, 10 minutes). The animals were incubated with the probes for 2.5 days at 55°C. Unbound probes were removed by five-minute washing steps with 100%, 75%, 50%, 25% hybridising solution in 2× SSC (0.3 M NaCl, 0.03 M sodium citrate in water, pH 7.0) followed by two incubations for 30 minutes in 2× SSC, 0.1% Chaps. The animals were equilibrated in maleic acid buffer (MAB: 100 mM maleic acid, 150 mM NaCl in water, pH 7.5) 2× for 10 minutes and blocked in 1% blocking reagent (Roche, Indianapolis, IN, USA) in MAB for two hours at room temperature. For detection of the DIG labelled RNA probes, an anti-DIG antibody coupled to alkaline phosphatase was used at 1:4000 in blocking solution at 4°C overnight. Unbound antibody was washed out during eight 30- to 60-minute washing steps with MAB, followed by an overnight washing step. To detect the signal the animals were first equilibrated 2× for 10 minutes in NTMT (100 mM NaCl, 100 mM Tris pH 9.5, 50 mM MgCl_2_, 0.1% Tween20) at room temperature and then incubated in NBT/BCIP (Roche, Indianapolis, IN, USA) 1:50 in NTMT in the dark at 37°C. When reaching the optimal signal to background ratio, the reaction was stopped by adding 100% ethanol. The animals were rehydrated by incubation for five minutes in 75%, 50% and 25% ethanol in 0.1× PBS. After a final rehydration step in PBS the animals were mounted on microscope slides in PBS 90% glycerol.

For double *in situ* hybridisation with Minicollagen-1 the animals were incubated simultaneously with DIG-labelled Cnidoin and fluorescein isothiocyanate (FITC)-labelled Minicollagen-1 probes. After incubation with the DIG antibody (1:2000) and MAB washing, the staining with NBT/BCIP was performed. The staining reaction was stopped with ethanol and after rehydration the animals were incubated with the FITC antibody (1:2000) overnight. After MAB washing, the minicollagen-signal was detected with the FastRed substrate (Roche). The reaction was stopped with 100 mM glycine, 0.1% Tween20, pH2.2. Samples were washed in PBS and mounted on microscope slides with PBS 90% glycerol.

### Microscopy

Fluorescence images were captured with the Nikon A1R confocal laser-scanning microscope (Nikon, Tokyo, Japan), in part at the Nikon Imaging Center, Heidelberg, Germany. *In situ* images were captured with the Nikon Eclipse 80i (Nikon, Tokyo, Japan).

### Transmission electron microscopy of recombinant Cnidoin

Supernatants of recombinant Cnidoin samples solubilised in 8 M urea were absorbed to freshly glow-discharged thin carbon films supported by thick perforated carbon layers and negatively stained with uranyl formiate following standard procedures [48].

### AFM force spectroscopy on recombinant Cnidoin monomers

Purified Cnidoin samples were stored at −80°C and thawed on crushed ice. Following the manufacturer’s protocol (Pierce, Rockford, Illinois, USA) samples were treated with TCEP for breaking inter- and intramolecular disulphide bonds and additionally with iodoacetamide for preventing their reformation.

Cnidoin constructs, bearing a C-terminal type I dockerin tag (GDVNDDGKVNSTDAVALKRYVLRSGISINTDNADLNEDGRVNSTDLGILKRYILKEIDTLPYKN) and an N-terminal ybbR tag (DSLEFIASKLA), were covalently attached to a functionalised glass slide displaying a coenzyme A (CoA) surface (Figure 4A). For functionalisation, glass slides were silanised with 3-aminopropyldimethylethoxysilane (ABCR) [49], coated with 25 mM N-hydroxy-succinimide (NHS)-PEG-maleimide (M = 425.39 g/mol) and treated with 25 mM CoA. Functionalised glass slides were incubated with 1 mg/ml of treated proteins in the presence of Sfp-transferase and MgCl_2_. After an incubation period of two hours, glass slides were carefully rinsed with Tris-buffered saline (25 mM Tris, 72 mM NaCl, pH 7.2), ensuring a permanent liquid environment for immobilised proteins and a loss of non-immobilised proteins. Silicon nitride AFM cantilevers bearing a silicon tip (BL-AC40TS-C2, Olympus, Center Valley, Pennsylvania, USA) were aminosilanised and coated with (NHS)-PEG-maleimide (M = 425.39 g/mol). Functionalised cantilevers were incubated with a 50 μM solution of CBM A2C-Cohesin (Addgene: pET28a-ybbR-HIS-CBM-CohI, 58709) [33] from *Clostridium thermocellum*, enabling specific binding to dockerin tagged proteins. All measurements were performed in Tris-buffered saline (25 mM Tris, 72 mM NaCl, pH 7.2) supplemented with 1 mM CaCl_2_ and 10 mM dithiothreitol (DTT) and protease inhibitor mix (Roche).

Single molecule force spectroscopy was performed using custom-built instruments [50] each driven by an MFP-3D AFM controller. Spring constants of the cantilevers were determined individually using the thermal noise method within 20% deviation of the nominal value of 0.07 N/m. Force-distance traces were recorded in closed-loop mode at pulling speeds ranging from 200 nm/s to 6,400 nm/s. With each trace the *xy*-stage was moved by 150 nm to probe a new position on the surface. For studying Cnidoin stretching, only retract traces with a final double peak (Figure 4B) were considered. This feature is characteristic of the cohesin-dockerin rupture [32] and guarantees that Cnidoin was stretched specifically from its C-terminus. A WLC model was fitted from zero to the first of the double peaks.

### Molecular dynamics simulations of Cnidoin’s repetitive sequence units

Two repetitive sequence units, namely *QMQGCGQQMPPMMSGCGG* and *QMQGCGQQLPLMMPGCVG*, were selected for investigation with MD simulations. Both of the units contain the *GCGQQ* motif and also a high content of methionine, two major features of Cnidoin (Figure 1C, Additional file 1: Figure S1B). Initial extended structures of these two units were constructed using Pymol [51] and were parameterised at the all-atom level resolution using the OPLS-AA force field [52].

All simulations were carried out using the MD software package Gromacs 4.5.3 [53]. The two Cnidoin peptide units with extended initial structures were first solvated in boxes of TIP4P water molecules with an ion concentration (Na^+^ and Cl^−^) of 0.1 mol/liter [54]. A cut-off of 1.0 nm was used for non-bonded interactions, and the Particle-Mesh Ewald method was chosen to sample long-range electrostatic interactions [55]. Periodic boundary conditions were employed to remove artificial boundary effects. In order to use a time step of 0.2 fs, all covalent bonds were constrained using the LINCS algorithm [56]. All simulations were performed under a constant temperature of 300 K and a constant pressure of 1 bar, using Nose-Hoover temperature coupling and Parrinello-Rahman pressure coupling methods, with coupling time constants of 0.4 ps and 4 ps, respectively [57-59]. The simulation systems were first energy minimised by using the steepest descent method. Equilibration of the solvent molecules was carried out for 500 ps, with all heavy atoms in the peptide restrained by a force constant of 1000 kJ · mol^−1^ · nm^−2^. The two Cnidoin units were then fully equilibrated for 500 ns individually. Energy and coordinates of the simulation systems were collected every 1,000 time steps. The same simulation parameters were used in the following simulations unless otherwise specified.

Umbrella sampling along the end-to-end distance of the two-peptide units was performed to probe their elasticity. Structures of the two units with shortest end-to-end distances in the above-mentioned 500 ns equilibration were chosen as starting points. Two new simulation systems comprising approximately 55,000 atoms, which were large enough to accommodate fully extended peptides, were set up. The peptides were extended by applying a pulling force as in force-probe MD simulations [60]. Peptide conformations covering Z-components of end-to-end distances between 0.4 and 7.0 nm were chosen as starting structures for the umbrella sampling runs, with distance intervals of 0.4 nm. A force constant of 500 kJ · mol^−1^ · nm^−2^ was used for the umbrella potential. The sampling times were changed with peptide extensions, with longer simulation times of 150 ns for extensions shorter than 2.0 nm because of higher fluctuations, and shorter times of 50 ns for longer extensions. The potential of mean force was calculated by using the weighted histogram analysis method [61]. Representative structures obtained from sampling at different extensions are shown in Figure 5A.

The dynamics of Cnidoin peptide collapse were investigated in an additional set of MD simulations. In these simulations, the two Cnidoin peptide units were first held by a constant pulling force of 415 pN at both termini for 20 ns to obtain a conformational ensemble with large end-to-end distances. We chose 10 of the stretched peptide conformations and initiated force-quench MD simulations in the absence of a pulling force to monitor peptide collapse. We defined structures with an end-to-end distance of 1.5 nm or smaller as the collapsed state, and measured the time each of the fully stretched peptides required to reach this state. For comparison, two disordered peptides from the sequence-related silk protein (Spidroin-1 and Spidroin-2) and, as a representative of a non-elastomeric protein, two disordered peptides from the vWF linker between domains A1 and A2, were chosen to perform the same collapse force-quench MD simulations (peptide sequences are shown in Additional file 7: Figure S7). Aiming at understanding the dynamical effects from the high percentage of methionine residues, for an additional set of simulations, all methionines in the Cnidoin peptides were mutated to alanines. The mutated Cnidoin peptides were then subjected to the same force-quench MD simulations.

### Molecular dynamics simulations of a bundle of Cnidoin’s repetitive sequence units

The repetitive sequence unit *QMQGCGQQLPLMMPGCVG* previously simulated in isolation was incubated with three other identical peptides spaced 2 nm along one of the Cartesian coordinates. Protein fragments were placed at the center of a cubic box with dimensions of 10 nm^3^. Simulation parameters and force fields were adopted from the simulations described above, if not otherwise mentioned. After a first energy minimisation the system was equilibrated in two steps. Firstly, temperature was coupled in a canonical (NVT) ensemble using a Nose-Hoover thermostat and secondly, pressure was coupled in an isobaric (NPT) ensemble using a Parrinello-Rahman barostat [57-59]. NVT and NPT equilibration runs were performed for 500 ps each, during which solvent molecules were able to accommodate around the protein that was fixed in space through the application of a 1,000 kJ · mol^−1^ · nm^−2^ force constant on each protein’s Cα atom. During the temperature-coupling step a set of initial, Boltzmann-distributed, velocities was generated. After equilibration, a 50 ns production molecular dynamics run was performed at the equilibrium, at a constant temperature of 300 K and a pressure of 0.1 bar. During such a run, the four filaments collapsed into a bundle subsequently used for the pulling simulations.

For the pulling simulations of a Cnidoin fragment embedded into a bundle of Cnidoin segments, the filament with the highest number of intermolecular contacts inside the bundle was chosen for pulling. The C-terminus Cα was set as pulling group whereas on the N-terminus Cα a positional restraint was placed using a force constant of 1,000 kJ · mol^−1^ · nm^−2^. The C-terminus Cα was pulled along the Z-component, as previously described, using an umbrella potential of 500 kJ · mol^−1^ · nm^−2^. Ten independent, pulling simulations were carried out.

### Fourier-transform mid-infrared spectroscopy

The degree of ordering was assessed on the basis of Fourier-transform mid-infrared spectroscopy of dried films of Cnidoin elastic domain. For recombinant expression of the Cnidoin elastic domain a DNA fragment coding for amino acids GGQM-AGCG was amplified by PCR and cloned four times as a tandem repeat into the pet21 vector (Novagen, Whitehouse Station, New Jersey, USA). Protein purification was performed under denaturing conditions via the C-terminal his-tag. For elution, buffer conditions were changed to PBS (136.9 mM NaCl; 2,7 mM KCl; 1.5 mM KH_2_PO_4_; 8 mM Na_2_PO_4_) including 10 mM DTT, 500 mM NaCl, 250 mM imidazole as well as protease inhibitor mix and the eluted protein was immediately used for the experiment. For comparison, nine further proteins (hemoglobin, bovine serum albumin, concanavalin, ribonuclease s, lysozyme, ferritin, cytochrome c, elastase, casein) were investigated in the same manner. Thus, nine different protein solutions at 1 mg/ml each were prepared in the elution buffer for Cnidoin elastic domain. Each solution was pipetted to 10 wells (50 μL per well) of a 96-well silicon sample carrier and left to dry. This procedure was repeated on three further sample carriers and each sample carrier was then investigated separately in order to check for consistency and reproducibility. Details of the technical setup for spectroscopy are described in [62]. After vector normalisation in the region from 1,600 cm^−1^ to 1,715 cm^−1^ and background subtraction, the median absorbance of each nine-fold replication on each sample carrier was calculated. In order to investigate the degree of ordering of the proteins we followed the implications of Byler and Susi [31] in that, both a low number of spectral components in the overall amide I band as well as the existence and strength of a peak around 1,645 cm^−1^ are indicative for a low degree of ordering. The number of spectral components was estimated from fitting one Gaussian curve per spectral component and comparing the fit results on the basis of the Akaike Information Criterion [63]. Median spectra were fitted with up to 13 Gaussian curves and up to 10 random starting conditions for any fixed number of Gaussian curves. An example of a median spectrum of Cnidoin is shown in Additional file 3: Figure S3C together with a fit result for five Gaussian curves (that is, five spectral components). All fit results were analysed by means of the corrected Akaike Information Criterion (AICc). Weights for the fit with i Gaussian curves were set to be $\frac{{{}^e}^{-\frac{AIC{c}_{\min^{-}}AIC{c}_i}{2}}}{{{}^{\sum_ie}}^{-\frac{AIC{c}_{\min^{-}}AIC{c}_i}{2}}}$ and the weighted arithmetic mean was calculated in order to yield the optimum number of Gaussians as well as its standard deviation.

For the investigation of peak positions and widths, those spectral components which contributed less than 1% to the total signal (area under the curve) were omitted from further analysis in order to avoid misleading conclusions. The peak around 1,645 cm^−1^ in the case of Cnidoin had an average width of 17.3 cm^−1^ and, mathematically, the difference from the widths of the other peaks is significant on the basis of a two-sided *t*-test.

However, despite the clear indications for the lower degree of ordering in Cnidoin, it has to be noted that many assumptions and simplifications enter into this analysis such that the results should be considered as supplementary information supporting the hypothesis of Cnidoin being a mainly unordered protein.

## Additional files

Additional file 1: Figure S1. Primary structure and amino acid composition of Cnidoin. (A) amino acid sequence and domain organization of Cnidoin. Signal peptide is in turquoise, propeptide in green, CRDs in yellow and the elastic domain in dark blue. Underlined is the second C-terminal CRD used as epitope for antibody generation. (B) amino acid composition of the elastic Cnidoin domain shows a high content in glycine, glutamine and methionine as well as cysteine and proline. Additional file 2: Figure S2. Co-polymerisation of Minicollagen and Cnidoin. Polymer products of recombinant Minicollagen-1-MBP as detected by Western blot are more pronounced with increasing amounts of recombinant Cnidoin. Additional file 3: Figure S3. (A) Disorder prediction of Cnidoin by DisEMBL. Disorder scores of loops and coils for each residue in Cnidoin are shown. Expected level of disorder is shown as orange dashed line. Repetitive sequence units in Cnidoin are highlighted by the light blue region. (B) Number of spectral components observed in the mid-infrared spectra of Cnidoin and other proteins. The red triangles represent the average number of components derived from multiple repetitions of the measurement procedure and the error bars indicate the standard deviation. Blue dots are the results obtained from Byler and Susi [31], from where it can be inferred that a low number of spectral components represents a low degree of ordering of the protein. (C) Example mid-infrared spectrum of Cnidoin together with a fit result for the case of five spectral components. Additional file 4: Figure S4. Forces of cohesin-dockerin rupture events as a function of the corresponding loading rates. For double rupture events, the ‘2nd’ denotes the final peak. In agreement with [32] forces are in the order of 100 pN and are highest for the second peak, followed by the first peak of double events, and are lowest for single rupture events. Additional file 5: Figure S5. (A) Force-distance traces of Cnidoin displaying only a final single peak. Double as well as single peak traces show identical characteristic worm-like chain (WLC) behaviour and lack further pronounced features. For quantification of contour and persistence length, a WLC model was fitted to single peak traces (red line). (B) Histogram of the contour length distribution of Cnidoin based on single peak traces. The average contour length of 97 nm and its corresponding standard deviation of 38 nm are in excellent agreement with average contour length (94 nm) and standard deviation (43 nm) for double peak curves (Figure 4C). (C) Histogram of the persistence length distribution of Cnidoin based on single peak traces. The average persistence length of 0.42 nm and its corresponding standard deviation of 0.21 nm are in excellent agreement with average persistence length (0.37 nm) and standard deviation (0.23 nm) for double peak curves. Additional file 6: Figure S6. Molecular elasticity of Cnidoin’s repeat engaging intermolecular contacts. The figure shows force-extension profiles for Cnidoin’s repetitive unit *QMQGCGQQLPLMMPGCVG* in the absence of other filaments in solution (red) and in the presence of four other identical fragments (black) as obtained from the average of 10 pulling simulations. The force-extension profiles have been fitted (solid lines) using a WLC model [36]. The obtained persistence length is 0.72 ± 0.02 nm for the single filament in water and 0.1 ± 0.003 nm for the filament embedded into the bundle. The snapshots at the top of the curves report the stretching of the pulled filament (orange) with respect to the bundle. The N-terminal position-restrained Cα is represented as a black sphere whereas the C-terminal pulled Cα is red. Filaments are represented as strings and atomic particles and bonds are shown using a balls and sticks representation and coloured by atom type. Additional file 7: Figure S7. Disordered peptide sequences studied beside Cnidoin peptide units. Each of the sequences used in this study has 18 residues as those in Cnidoin peptides. Mutation points in Cnidoin peptides from methionine to alanine are highlighted in red in the figure.
