# Supplementary figures and images for: A fast recoiling silk-like elastomer facilitates nanosecond nematocyst discharge
Source: BMC Biol. 2015 Jan 16;13:3. doi: 10.1186/s12915-014-0113-1 (PMC4321713; doi:10.1186/s12915-014-0113-1)

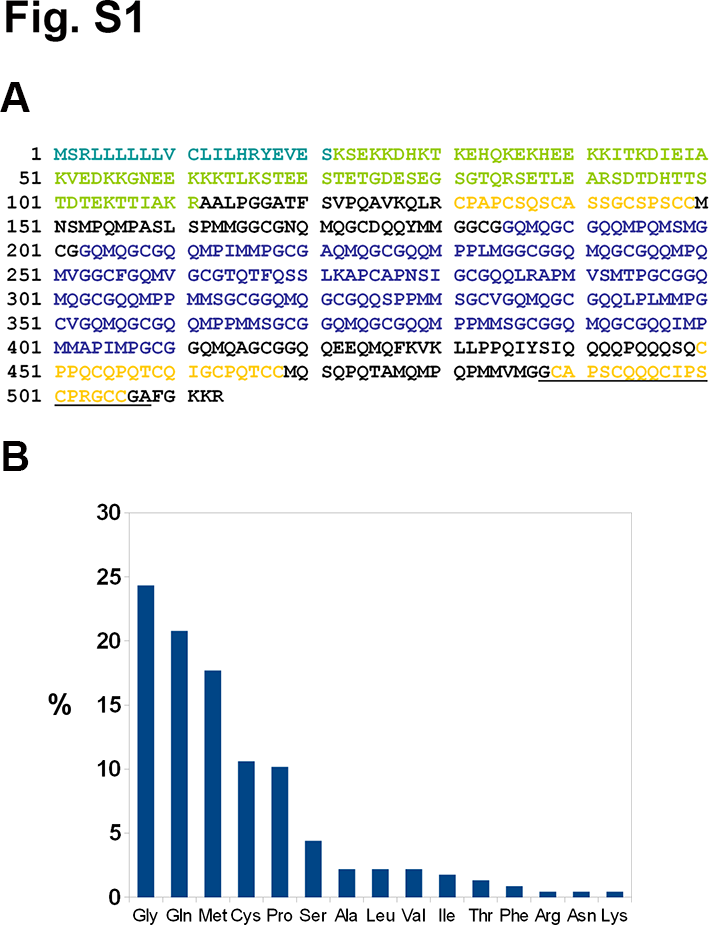

Supplement: Additional file 1: Figure S1. — Primary structure and amino acid composition of Cnidoin. (A) amino acid sequence and domain organization of Cnidoin. Signal peptide is in turquoise, propeptide in green, CRDs in yellow and the elastic domain in dark blue. Underlined is the second C-terminal CRD used as epitope for antibody generation. (B) amino acid composition of the elastic Cnidoin domain shows a high content in glycine, glutamine and methionine as well as cysteine and proline. [file 12915_2014_113_MOESM1_ESM.tiff]

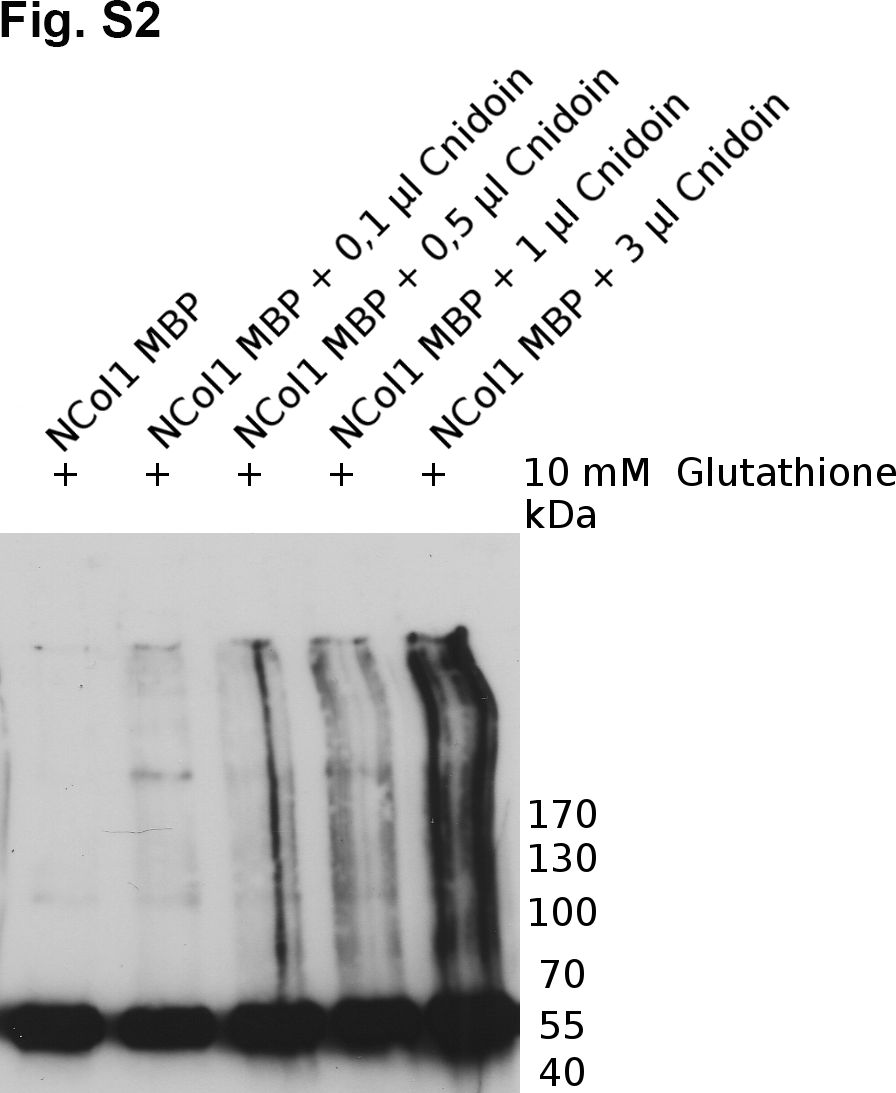

Supplement: Additional file 2: Figure S2. — Co-polymerisation of Minicollagen and Cnidoin. Polymer products of recombinant Minicollagen-1-MBP as detected by Western blot are more pronounced with increasing amounts of recombinant Cnidoin. [file 12915_2014_113_MOESM2_ESM.tiff]

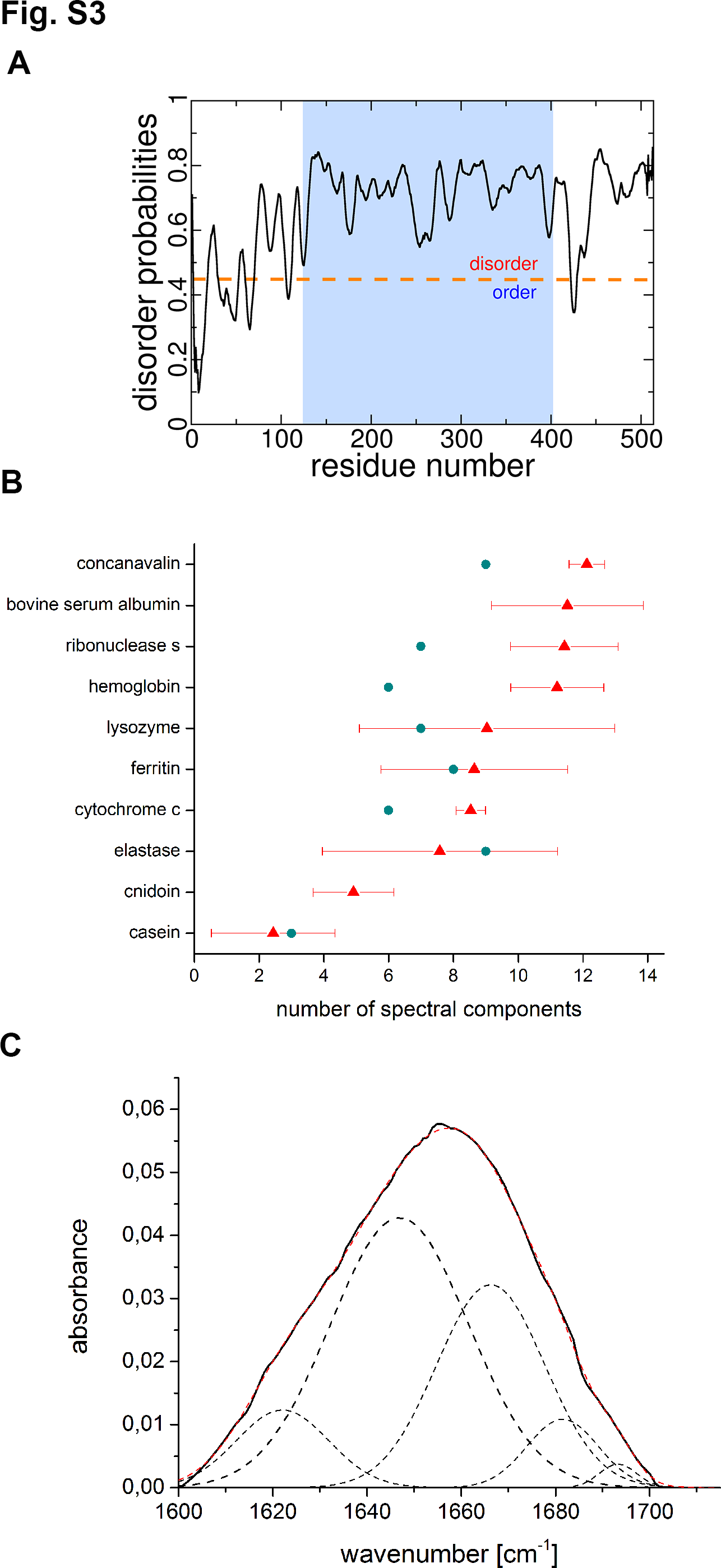

Supplement: Additional file 3: Figure S3. — (A) Disorder prediction of Cnidoin by DisEMBL. Disorder scores of loops and coils for each residue in Cnidoin are shown. Expected level of disorder is shown as orange dashed line. Repetitive sequence units in Cnidoin are highlighted by the light blue region. (B) Number of spectral components observed in the mid-infrared spectra of Cnidoin and other proteins. The red triangles represent the average number of components derived from multiple repetitions of the measurement procedure and the error bars indicate the standard deviation. Blue dots are the results obtained from Byler and Susi [31], from where it can be inferred that a low number of spectral components represents a low degree of ordering of the protein. (C) Example mid-infrared spectrum of Cnidoin together with a fit result for the case of five spectral components. [file 12915_2014_113_MOESM3_ESM.tiff]

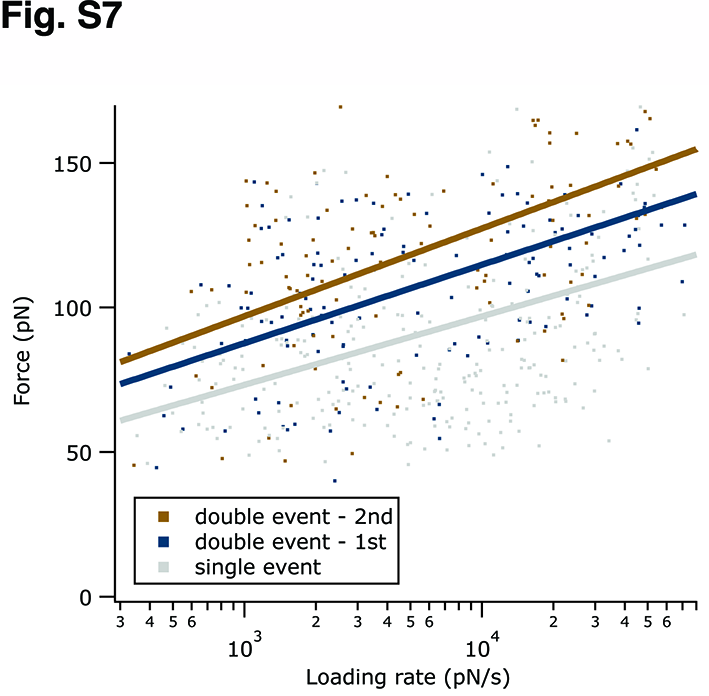

Supplement: Additional file 4: Figure S4. — Forces of cohesin-dockerin rupture events as a function of the corresponding loading rates. For double rupture events, the ‘2nd’ denotes the final peak. In agreement with [32] forces are in the order of 100 pN and are highest for the second peak, followed by the first peak of double events, and are lowest for single rupture events. [file 12915_2014_113_MOESM4_ESM.tiff]

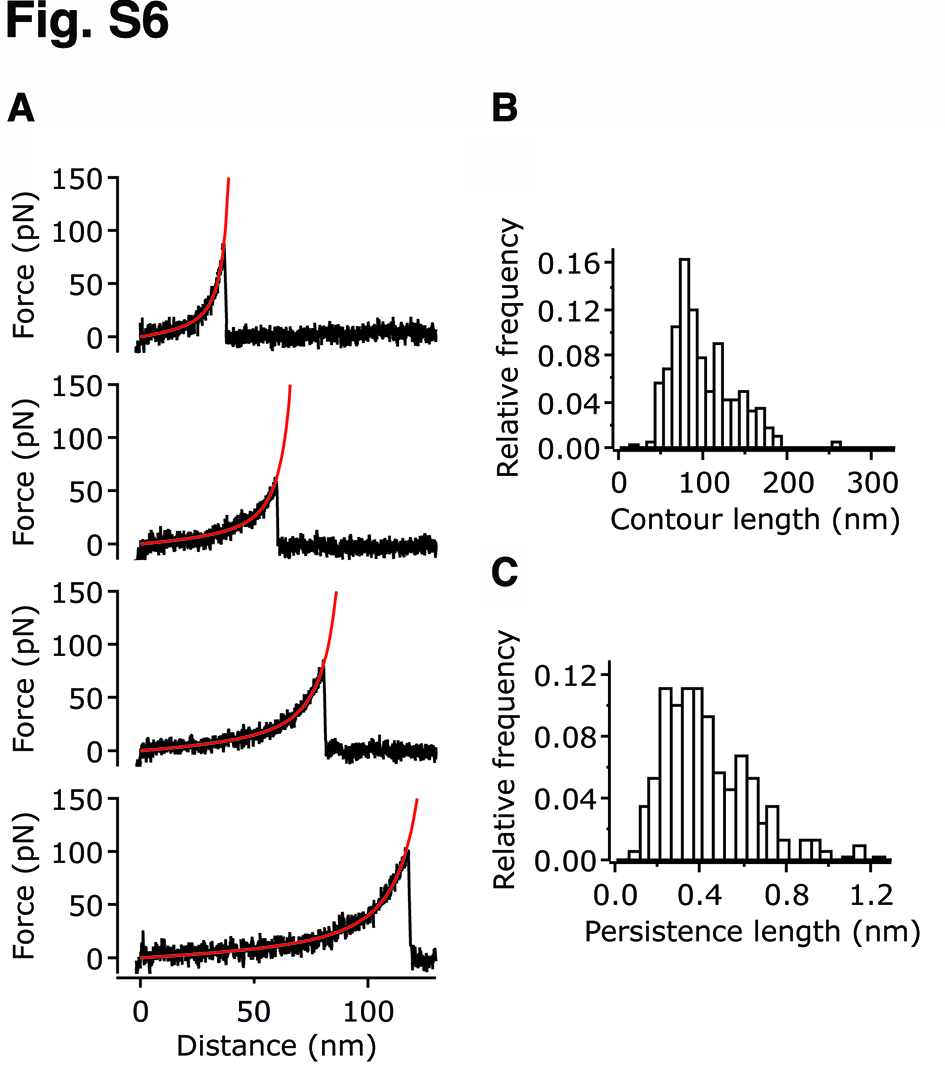

Supplement: Additional file 5: Figure S5. — (A) Force-distance traces of Cnidoin displaying only a final single peak. Double as well as single peak traces show identical characteristic worm-like chain (WLC) behaviour and lack further pronounced features. For quantification of contour and persistence length, a WLC model was fitted to single peak traces (red line). (B) Histogram of the contour length distribution of Cnidoin based on single peak traces. The average contour length of 97 nm and its corresponding standard deviation of 38 nm are in excellent agreement with average contour length (94 nm) and standard deviation (43 nm) for double peak curves (Figure 4C). (C) Histogram of the persistence length distribution of Cnidoin based on single peak traces. The average persistence length of 0.42 nm and its corresponding standard deviation of 0.21 nm are in excellent agreement with average persistence length (0.37 nm) and standard deviation (0.23 nm) for double peak curves. [file 12915_2014_113_MOESM5_ESM.tiff]

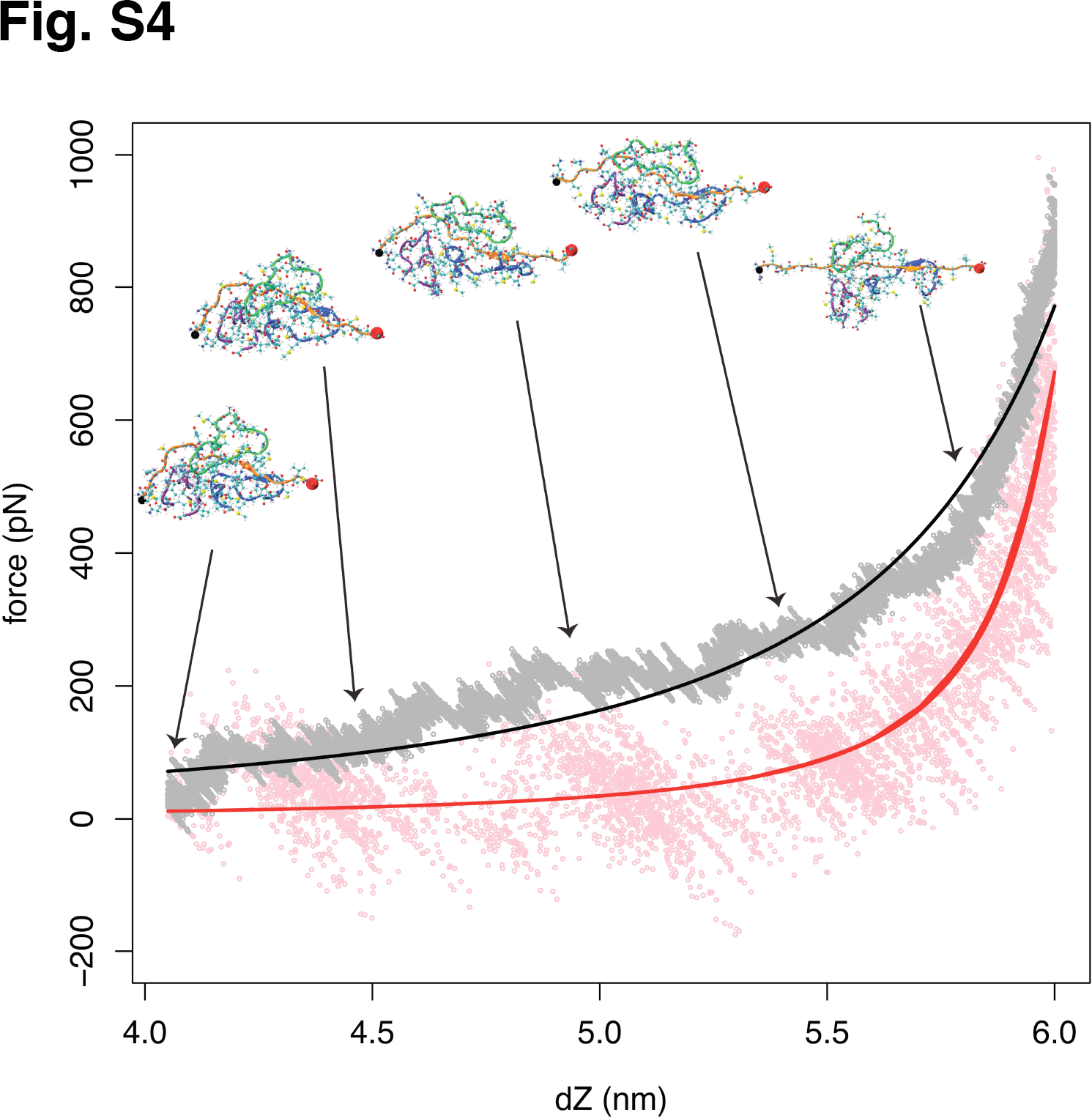

Supplement: Additional file 6: Figure S6. — Molecular elasticity of Cnidoin’s repeat engaging intermolecular contacts. The figure shows force-extension profiles for Cnidoin’s repetitive unit QMQGCGQQLPLMMPGCVG in the absence of other filaments in solution (red) and in the presence of four other identical fragments (black) as obtained from the average of 10 pulling simulations. The force-extension profiles have been fitted (solid lines) using a WLC model [36]. The obtained persistence length is 0.72 ± 0.02 nm for the single filament in water and 0.1 ± 0.003 nm for the filament embedded into the bundle. The snapshots at the top of the curves report the stretching of the pulled filament (orange) with respect to the bundle. The N-terminal position-restrained Cα is represented as a black sphere whereas the C-terminal pulled Cα is red. Filaments are represented as strings and atomic particles and bonds are shown using a balls and sticks representation and coloured by atom type. [file 12915_2014_113_MOESM6_ESM.tiff]

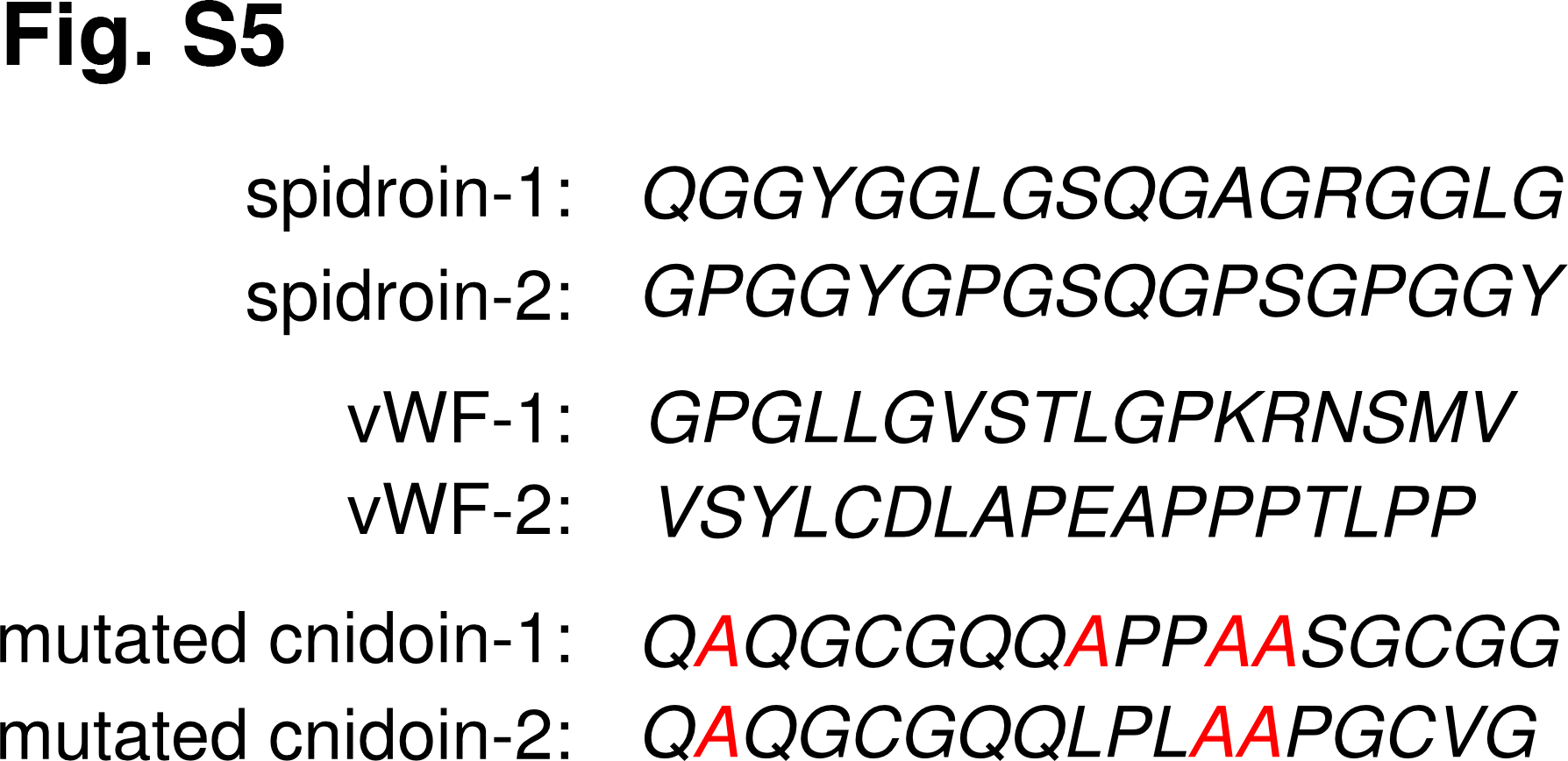

Supplement: Additional file 7: Figure S7. — Disordered peptide sequences studied beside Cnidoin peptide units. Each of the sequences used in this study has 18 residues as those in Cnidoin peptides. Mutation points in Cnidoin peptides from methionine to alanine are highlighted in red in the figure. [file 12915_2014_113_MOESM7_ESM.tiff]
